# Caring animals and care ethics

**DOI:** 10.1007/s10539-022-09857-y

**Published:** 2022-05-26

**Authors:** Birte Wrage

**Affiliations:** Unit of Ethics and Human-Animal Studies, Messerli Research Institute, Vetmeduni Vienna, Uni Vienna, MedUni Vienna, Vienna, Austria

**Keywords:** Animal morality, Empathic care, Care ethics, Parental care, Moral emotions

## Abstract

Are there nonhuman animals who behave *morally*? In this paper I answer this question in the affirmative by applying the framework of care ethics to the animal morality debate. According to care ethics, empathic care is the wellspring of morality in humans. While there have been several suggestive analyses of nonhuman animals as empathic, much of the literature within the animal morality debate has marginalized analyses from the perspective of care ethics. In this paper I examine care ethics to extract its core commitments to what is required for moral care: emotional motivation that enables the intentional meeting of another’s needs, and forward-looking responsibility in particular relationships. What is not required, I argue, are metarepresentational capacities or the ability to scrutinize one’s reasons for action, and thus being retrospectively accountable. This minimal account of moral care is illustrated by moral practices of parental care seen in many nonhuman animal species. In response to the worry that parental care in nonhuman animals lacks all evaluation and is therefore nonmoral I point to cultural differences in human parenting and to normativity in nonhuman animals.

## Introduction

Are there nonhuman animals who can be credited with morality? The empirical strand of the animal morality debate points to the existence of (proto-)moral capacities such as empathy, altruism, and inequity aversion, and prosocial behaviors like helping, consolation, fair-play, and cooperation in a range of nonhuman animal species (for discussions see de Waal 1996; Bekoff & Pierce 2009; Vincent et al. 2019; Andrews 2020b, ch. 9). The philosophical strand of the debate on morality in nonhuman animals, however, struggles to affirm these observations as evidence of morality, because morality is traditionally conceived of in anthropocentric and highly intellectualistic terms. Much of the Western ethical tradition has taken morality to be based on the ability to understand and scrutinize moral rules, principles and duties, which limits it to humans. Reflection on one’s moral motivation, which involves metacognition, is thought to be a crucial feature of morality. This leaves the debate on nonhuman animal morality at an impasse: On the one hand, we have what some take as compelling empirical evidence of behavior that deserves the label ‘moral’ in nonhuman animals. A core argument here is to evaluate human and nonhuman behavior by the same standards. On the other hand, our philosophical concepts of morality fail to account for morality in nonhuman animals, because they assume human superiority.

Some headway has been made towards acknowledging morality in nonhuman animals by scientists and philosophers who argue for the de-intellectualization of these traditional Western notions of morality (e.g. Waller 1997; Bekoff & Pierce 2009; Rowlands 2012; Monsó 2015; 2017; Monsó et al. 2018; Rutledge-Prior 2019; Behdadi 2021; Monsó & Wrage 2021). They argue for minimal accounts of moral agency that do not require moral judgement as metarepresentational scrutiny of reasons for action. Some accounts instead acknowledge *emotionally* motivated behavior as potentially moral and suggest we turn our focus to empathy as a moral motivation (Waller 1997; Rowlands 2012; Andrews 2013; Andrews & Gruen 2014; Monsó 2015; 2017). In this paper, I propose that instead of making traditional moral theories bend to this de-intellectualized concept of morality to include nonhuman animals, there is a more direct way to move the animal morality debate forward: Care ethics as a sentimentalist moral theory acknowledges moral emotions and situates morality in particular relationships, instead of in the realm of abstract and impartial reason. It may thus be more open to the inclusion of nonhuman animals as moral agents.

Care ethics is an ethical framework that claims that care, or the *intentional*^1^*meeting of another’s needs*, is central to morality. In this framework emotions such as empathy and sympathy play the main role in moral motivation; paradigmatically, we provide care for others because we care about them emotionally (e.g. Noddings 1984/2013, 9f; Tronto 1993, 102f; Held 2006, 30). Moreover, care ethicists argue that care is not just one among many moral practices, but that caring relationships are the biological root of any moral concern (e.g. Baier 1987/2002). This argument regarding the biological origins of morality provides a straight-forward opportunity to talk about morality in nonhuman animals. However, care ethics cannot be applied to nonhuman animals ‘off the shelf,’ because it is situated in a larger anthropocentric framework. Thus, I will examine care ethics and extract what I take to be its core requirements for moral care: emotional motivation that enables the intentional meeting of another’s needs, and forward-looking responsibility in particular relationships. What is not required, I argue, are metarepresentational capacities or the ability to scrutinize one’s reasons for action, and thus being retrospectively accountable, which would exclude (most) nonhuman animals. I illustrate this minimal account of moral care by considering parental care in many nonhuman animal species.

The structure of the paper is as follows: In Sect. 2, I review how care ethicists understand moral care. I identify the claim that moral care requires second-order reflection as an obstacle to the inclusion of caring animals, and point out potential pitfalls of this standard. I propose to widen this account to make it more inclusive of human as well as nonhuman animal practices of care that are emotionally motivated in Sect. 3. Moral care in a care ethics framework, I propose, minimally consists of empathically motivated meeting of another’s needs and being prospectively responsible in relationships. I use the terms ‘emotional care’ or ‘empathic care’ to refer to this nonreflective form of care. Section 4 reviews the empirical data on this care in nonhuman animals, focusing on parental care. I conclude that many nonhuman animals can be credited with moral care thus understood, and give an outlook on possible ethical implications in Sect. 5.

On this note, before I begin, I want to acknowledge that some of the research I cite in this paper to illustrate nonhuman animals’ caring capacities is ethically problematic, because it lacks respect for caring animals as such and for their relationships. I refer to this research with regret and in hopes that my argument inspires care ethical criticism of this lack of care for caring animals.

## Moral care

In this section, I give a brief introduction to care ethics and summarize how care ethics understands moral care, or ‘what it takes’ to participate in care as a moral practice. I then propose to widen this account to make it more inclusive of human practices of care as well as care in nonhuman animals. I mainly refer to foundational texts of care ethics, as these texts established what it means to be a moral caregiver. These standards are the relevant frame of reference in arguing for moral care in nonhuman animals, although they were developed with humans in mind, sometimes even in explicit distinction from other animals. Moreover, as I write this paper as a contribution to the interdisciplinary debate on nonhuman animal morality, I limit myself to a few main positions in care ethics to give an overview.

I view the care ethical literature focused on nonhuman animals as supplementary here. This is because it mostly focuses on the moral standing of nonhuman animals as *recipients of human caring*, not on nonhuman animals’ own *agency* in the practice of care (see Donovan & Adams 1996; 2007,2). An exception is Gruen (2015), who points out the value of caring relationships that do not involve humans in chimpanzees, and thus hints at nonhuman animals’ own ‘entanglement’ in their intraspecific relationships of care. Indeed, an important upshot of my argument for moral care in nonhuman animals is the respect that humans can be argued to owe to caring animals and their relationships. However, considering these ethical implications is, unfortunately, beyond the scope of this paper.

### Moral care according to care ethics

Care ethics is a relatively young moral theory that emerged as a critical response to traditional moral philosophy in the 1980s. Feminist moral philosophers began to criticize the traditional focus on the entitlements of autonomous agents and argued for a more relational approach to morality that revolves around care and responsibility. Care ethicists view care as a *moral practice* that is fundamental to morality. Care is a practice in that paradigmatic care is not just an internal state or attitude, *caring about*, but entails behavior that is motivated by this internal state, *caring for* (Noddings 1984/2013, 9f; Tronto 1993, 102f; Held 2006, 30). This practice is an integral part of human social lives, and parental care has a special place in this practice, as it is crucial for children’s social development (e.g. Feldman 2011). We need good parental care to become capable of being invested in others’ wellbeing, and thus become moral beings. Therefore, care is fundamental to a moral society in two ways: as a continuous practice that responds to others’ needs, and as a precondition for being able to engage in this practice.

In its conception of care as a moral practice, care ethics decidedly centers on real life and aims to capture everyday care, not the exceptional. Within this everyday care, care ethicists acknowledge emotion as a valid (part of) moral motivation (e.g. Baier 1987/2002, 236; Held 1993, 52). In fact, familial relationships and the entailed emotional attachments are taken to be the wellspring of moral concern and the paradigm of moral care by care ethicists: These particular relationships, Baier (1987/2002, 236) explains, “[give] rise to moral obligations more self-evident than any obligation to keep contracts.” The moral obligations that emerge in close relationships are so self-evident, so compelling, precisely because they stem from sentiment instead of pure reason. Accordingly, Held (1993, 52) writes, care ethics “will embrace emotion as providing at least a partial basis for morality itself.” With this acknowledgment, care ethics stands in the sentimentalist tradition. Sentimentalists such as Hume, Baier points out, “[endorse] the emotional response to a fully realized situation as moral reflection at its best, not as one of its underdeveloped stages” (Baier 1987/2002, 239).

The emotions care ethicists usually refer to as adequate motivation for care are sympathy (Noddings 1984/2013, ch. 2; Baier 1987/2002; Collins 2015, 23ff) or empathy (Held 2006; Slote 2007). Although each author gives reasons for preferring one over the other, and there is a larger debate on the distinctions between them, for space reasons I will focus on the common ground: the emotion motivating care is crucially a ‘feeling with the other’ that centers their needs. For the sake of simplicity, and because I want to bring empirical data on care in nonhuman animals into this debate, I will henceforth use the term ‘empathy’ to refer to the emotional motivation that is crucial in care ethics.^3^ Empathy is generally a heterogenous concept that can be understood in highly intellectualistic terms to involve Theory of Mind (e.g. considering another’s mental states in comparison to one’s own), projection (putting oneself in the other’s shoes) or other forms of second-order reflection (Decety et al. 2016, 2). However, it can also be understood in less intellectualistic terms. For instance, emotional contagion, which is widely regarded as a minimal form of empathy (de Waal 2008; Decety et al. 2016; Bartal et al. 2011), merely involves the involuntary ‘catching’ of another’s emotion, and does not involve any cognitively complex capacities like perspective-taking (Meyza & Knapska 2018).

Care ethicists tend to occupy a middle ground between these two poles in their understanding of empathy, and emphasize direct perception (e.g. Noddings 1984/2013, ch. 2), attentiveness (e.g. Tronto 1993, 127f), and responsiveness (e.g. Tronto 1993, 134f; Held 2006, 15; ibid., 24) over projection as central features of empathic care. In fact, some care ethicists argue that projective perspective-taking without actual ‘feeling with the other’ bears the risk of overshadowing the other’s actual needs and is thus less desirable (Noddings 1984/2013 ch. 2; Gruen 2015, 56f). Crucially, empathy must be about the other, not about oneself, and this mark can be missed with a cognitively ‘too primitive’ as well as a ‘too sophisticated’ form of empathy. Capturing this desired blend of emotional involvement and other-directedness, Noddings (1984/2013, 16ff) calls the state that caring ideally induces in the caregiver “engrossment” or “motivational displacement.”

This is a notion of empathy that may not necessarily require metarepresentation (more on this in subsection 3.1), however, care ethicists hold that care must involve some degree of reflection to count as moral. While emotion is argued to be at the root of morality, rational reflection refines this emotion, as it enables one to question the adequacy of care. Held (2006, 10) holds, for instance, that “raw emotion” cannot be a guide to morality, “feelings need to be reflected on and educated.” Moreover, care needs to be “subjected to moral scrutiny and *evaluated*” (ibid., 11, emphasis in original). This ‘moral judgment’ is intended to help avoid misguided or misplaced care, since purely emotional care is at risk of becoming excessive or controlling (ibid., 11), and is unreliable as a moral compass. Baier (1987/2002, 230), referencing Hume again, calls the core moral capacity “corrected sympathy,” i.e. an emotional motivation that has been reflected on, and understands mere emotional motivation as a *proto-*moral capacity (ibid., 246-7). Ruddick (1980, 347) calls the crucial blend of emotion and reflection in what she takes to be paradigmatic moral care “maternal thinking,” which is informed by “the intellectual capacities [one] develops, the judgments [one] makes, the metaphysical attitudes [one] assumes, the values [one] affirms.” In her introduction, Noddings (1984/2013, 3) even promises not to “bog down in sentiment.” Thus, while emotion is at the root of morality according to care ethics, it is not enough for fully moral behavior but needs to be reflected on.

From this rough summary we can discern that there is ground for hoping that empathic care in nonhuman animals could be acknowledged as moral by care ethics, but there are also hindrances to this proposition. On the one hand, promisingly, the evolutionary account of morality that is implied in arguments regarding the origin of morality in affective familial relationships is open to arguments for morality in other social species. Many nonhuman animals have, as I will show, strong familial bonds and provide extensive parental care. If this setting is where morality emerges in humans, this is also where we could find morality in nonhuman animals. Moreover, care ethicists see emotionally motivated care as the paradigmatic form of care. Care ethics’ relatively modest definition of empathy, and its situatedness in particular relationships instead of universal abstractions suggest a concept of moral care that is in principle attainable for nonhuman animals. On the other hand, however, care ethicists do insist on varying degrees of reflection, sometimes second-order thought, as a corrective to emotionally motivated care, which sets the cognitive bar too high for nonhuman animals. However, I think this intellectualization already causes tension in human-centered care ethics, because it seems to backpedal on the recognition of emotion as moral motivation that care ethicists themselves base their entire case on. In the next section I will therefore consider the cost of this intellectualization of care that ties morality to metacognition.

### Pitfalls of making moral care reflective

While its fundamental reliance on emotions makes care ethics potentially inclusive of caring animals, its later recourse to second-order reflective thinking excludes (most of) them. Granted, reflective care may make for salient examples of moral care. However, this narrow account of moral care risks reducing morality to its most rarefied form even in humans and ignoring the morality of everyday social interactions. Andrews and Gruen (2014, 209) write:

Once we are able to look past the most salient examples of human morality, we find that moral behavior and thought is a thread that runs through our daily activities, from the micro-ethics involved in coordinating daily behaviors like driving a car down a crowded street (Morton 2003), to the sharing of someone’s joy in getting a new job or a paper published. If we ignore these sorts of moral actions, we are overintellectualizing human morality…

Care ethics criticizes this reduction of morality to the extraordinary when it points out that the abstract moral realm concerned with justice and autonomous agents is impossible without the ubiquitous moral groundwork of care that produces moral beings in the first place (Held 1993, 55). However, it risks reproducing this overly narrow view of morality if it ties the morality of care to a capacity that neurotypical adult humans generally possess, but may not typically utilize in their caring. If our moral behaviors do not *typically* involve metacognition, it is unclear why these atypical instances should be hailed as the benchmark of morality, instead of as merely one moral mode among others.

Reflection can help make up for lack of experience or when we consciously want to change how we care, but the goal seems to be to become, in fact, someone who can provide care spontaneously. It would thus be undesirable for care ethics to limit moral care to reflective care. In many cases, we simply do not need second-order reflection to provide adequate care. This is also because the adequacy of care not only hinges on the wider, oftentimes abstract context, but is something the care recipient can give direct feedback on, which is why empathic capacities that make someone sensitive to others’ emotional state are so crucial for care, unlike reflection. In fact, in some cases, “the moral lustre is tarnished if deliberation intervenes,” as Waller (1997, 344) puts it. Having “one thought too many” (Williams 1981, cited after Wolf 2012) is worse than having one thought too little when it comes to care, because the latter, despite being erroneous, still speaks to a good moral character.

## Minimal moral care

If we give up reflection as the criterion for morality, what are the minimum capacities required for care so that we can still call it moral care in a care ethics framework? There are two authors outside the care ethical tradition I am aware of who have made some suggestions. First, Rowlands (2012, 38, endnote 14) mentions that his de-intellectualized notion of morality as the right kind of nonreflective emotional motivation without moral responsibility is “consistent” with “some versions of care ethics.” However, Rowlands does not elaborate on this. Second, Waller (1997) sees care ethics as a framework that points to the virtuousness of ‘nonreflective’ intentional behaviors and thus to nonhuman animal morality. Yet, he does not identify obstacles within care ethics to this view, as his focus is on the exclusion of nonreflective moral behaviors from *traditional* accounts of morality. Moreover, neither of the two authors connects this to parental care in nonhuman animals or considers possible ethical implications. I hope to add to these accounts here.

Based on Rowlands and Waller, I propose that the question of moral care hinges on whether a creature *intentionally*^4^*engages in care based on the right motives*. In addition, unlike Rowlands and Waller I think we need to ask whether the care ethical notion of *responsibility* can be applied to these caring creatures in a meaningful way. I take this as the minimal standard for what it means to fully participate in the moral practice of care. In the following subsections I thus argue for nonreflective empathy as ‘the right kind of motivation’ for care (3.1), and show that nonreflective caring animals can still be responsible in a sense that is relevant for care ethics (3.2). Taken together, this provides a de-intellectualized account of moral care that is consistent with care ethics despite being nonreflective. I use the term ‘nonreflective’ as a shorthand for ‘not involving metacognition,’ not to mean that there is no cognition involved, as I do understand emotion to involve cognition. I also address two objections from care ethicists against the continuity of moral caring capacities in nonhuman animals (3.3).

### Nonreflective empathy

Empathy is noted as a core emotional motivation for care by care ethicists. In this subsection I specify the concept of empathy that is sufficient as a moral motivation for minimal moral care. This is a concept of empathy that does not require metacognition and is in line with the care ethical understanding of empathy as a ‘feeling with the other’ that is other-directed instead of self-directed (see Sect. 2). Moreover, as I have noted above, care ethicists view the direct perception of the other’s utterances and behavior as crucial for the caregiver’s empathic process. Monsó (2017) makes the case for a “minimal moral empathy (MME)” (ibid., 215) as a moral emotion that matches these ideas, and which I will therefore apply here.

Care ethics already refers to moral emotions as *part of* moral motivation, but I think they can serve as moral motivation on their own. I understand moral emotions the following way: Moral emotions have evaluative content regarding a morally relevant feature of the world, and are experienced as the result of a reliable normative sensitivity, a “sensitivity to the good- and bad-making features of situations,” such as others’ happiness or suffering (Rowlands 2012, 230). Examples of (positive and negative) moral emotions besides empathy would be gratitude, jealousy, schadenfreude, or cruelty. Crucially, these emotions have a moral content whether or not the being experiencing them can intellectually entertain a relevant moral proposition such as e.g. ‘Your suffering is a good thing’ (schadenfreude), because moral emotions “track” moral propositions (ibid., 58ff). Tracking here denotes an asymmetric truth-preserving relation between moral propositions and moral emotions (ibid.). Following Rowlands (ibid.), a moral emotion *tracks* a moral proposition if the truth of said proposition guarantees the truth of said moral emotion “in virtue of the fact that there is a reliable asymmetric connection between the concepts expressed by [said proposition] and the concept expressed by [said moral emotion].” For instance, schadenfreude as ‘joy elicited by another’s suffering’ tracks the moral proposition “Your suffering is a good thing.” A being need not entertain the latter proposition to experience schadenfreude and thus to behave morally motivated by it. They only need the capacity to recognize that another is suffering, and revel in it. Thus, whenever someone acts motivated by such nonreflective moral emotions, they act morally, although they are not intellectually entertaining any relevant moral proposition themselves.

On the background of this definition by Rowlands (2012), Monsó (2017, 350) defines MME the following way:

Creature C possesses minimal moral empathy (MME) if: (1) C has an ability to detect distress behaviour in others, and (2) due to the action of a reliable mechanism, the detection of distress behaviour in others results in a process of emotional contagion that (3) generates a form of distress with the other’s distress behaviour as its intentional object, built into which is (4) an urge to engage in other-directed affiliative behaviour.

Conditions 1 and 2 constitute the *normative sensitivity to a bad-making feature of a situation*, conditions 3 and 4 are the features of MME that grant that it ‘tracks’ the moral proposition “This creature’s distress is bad” (ibid., 351). MME is intentional, albeit in a nonreflective sense (condition 3): A being with MME is not just distressed *when* another is distressed, but distressed *that* another is distressed (Monsó 2017, 348). This aboutness of the distress experienced via emotional contagion is evident in the motivation to do something to alleviate it *in the other*, which leads to other-oriented care behavior (e.g. consolation or helping behavior), instead of e.g. removing oneself from the situation to alleviate self-directed distress (ibid., 351). Thus, the other’s distress becomes a *reason*^5^ for care behavior (ibid.).

This minimal notion of empathy is highly compatible with the focus care ethicists have put on attentive behavior-reading over projective mindreading in their definition of empathy, and with the paradigmatic understanding of care as involving ‘caring about’ and ‘caring for,’ as MME entails the urge to care. Moreover, MME can be based on mere emotional contagion, the spontaneous catching of another’s affect (Meyza & Knapska 2018), as a reliable mechanism to be affected by another’s distress. Although emotional contagion is widely regarded as merely a basis for or the simplest form of empathy, the other conditions of MME ensure that we are speaking of a form of empathy that is actually intentional and other-directed.

### Nonreflective responsibility

I have argued that we can renounce reflection on one’s motives, and instead rely on empathy to morally motivate care. Thus, empathic animals without the capacity for second-order reflection on their motives can care morally. Without this capacity for second-order reflection one is not morally responsible in the traditional sense, namely, one cannot be held retrospectively accountable, i.e. be praised or blamed. However, responsibility is a core maxim of care ethics. How do we reconcile this? In the animal morality debate it has been suggested that the question whether a creature can behave morally is independent of the question whether they can be morally responsible (e.g. Waller 1997; Rowlands 2012). This could be applied to the morality of care, but I suspect this approach will be unappealing for care ethicists. It is precisely the achievement and merit of care ethics to shine a light on our moral obligations beyond those that are traditionally centered, namely our ‘special obligations’ (Walker 2007, 83). Responsibility for particular others as a result of and response to dependence is its overarching maxim (Collins 2015). However, we shouldn’t prematurely assume that this notion of responsibility necessarily relies on second-order reflection and is thus beyond the reach of nonhuman animals.

Responsibility according to care ethics means being responsive to others’ needs and continuously tending to one’s relationships; it is a caring response to factual dependence (e.g. Tronto 1993, 79ff; Held 2006, 10ff; see also Collins [2015, 88ff] for an overview of notions of responsibility in care ethics). This responsibility is primarily prospective and towards particular others, instead of retrospective and universal/abstract, and has also been understood as responsiveness, attentiveness, or caring perception (Tronto 1993, 127ff; Gruen 2015, 3; ibid., 34). Responsibility thus understood is an inherent feature of caring relationships, because by building and maintaining relationships of care, one *is* behaving responsibly. Caring animals are, thus, also responsible animals.

### Two objections: empathic care is automatic and lacks evaluation

There are two instances I am aware of where care ethicists have made an explicit effort to *exclude* caring animals, which I want to respond to here. If caring affect in familial relationships is the biological root of moral concern (see especially Noddings 1984/2013, ch. 4; 2010; Baier 1987/2002; Waller 1997), a crucial implication is the issue of phylogenetic continuity, i.e. that this moral capacity may be found in other species. Noddings and Held do acknowledge this continuity between the caring capacities of human and nonhuman animals, but argue that something still sets human caring apart: Noddings (1984/2013, ch. 4) distinguishes between *nonmoral* “natural care,” i.e. empathic care, and “ethical care,” which combines the emotional motivation seen in natural care with a reflective affirmation of that motivation. For Noddings, reflection is not primarily a corrective, but linked to a recognition of duty, which she admits to be a Kantian notion of morality (ibid., 80). To her, ‘ethical caring’ is meant as a failsafe for those situations where we do not care naturally, but *should* (ibid., 81f). Since nonhuman animals lack the capacity to reflectively motivate their care in absence of an emotional motivation, she concludes that their care is nonmoral (ibid., 79). However, by introducing this distinction we do not yet get an argument why ‘natural care’ would be nonmoral; it just contrasts it with a form of moral care that is salient when framed in a traditional understanding of morality.

Held, in turn, argues that human caring overcomes its ‘naturalness’ and sets humans apart from caring animals, because human parents (consciously) educate their children morally:

Human mothering is a far different activity from the mothering engaged in by other animals …. Human mothering shapes language and culture, it forms human social personhood, *it develops morality*. Animal behavior can be highly complex, but it does not have built into it any of the *consciously chosen aims of morality*. In creating human social persons, human mothering is different in kind from merely propagating a species. (Held 1993, 55, my emphasis)

I see two interrelated potential problems with this response: It overemphasizes similarities across human cultural practices and underemphasizes similarities between humans and nonhuman animals. Held gives a rather narrow depiction of human parenting that best matches the fairly intellectualized parenting that has only relatively recently become necessary in increasingly individualistic cultures^6^, and that, in these contexts, some have privileged access to. The singular mother appears as the sole source of parental care, including moral education. Parents in more traditional communitarian cultures and societal classes with closer community support, however, may trust and rely on the knowledge of more experienced parents. Hrdy (2009, ch. 3) argues this to be the (pre-)historically older and more widespread version of parenting, because our hunter-gatherer ancestors could not have survived without relying on alloparenting, on a community of many parents, to raise infants. In such a context, where parenting does not constantly have to be ‘re-invented,’ the commitment to any “aims of morality” may be less explicit than Held (1993, 55) makes it sound. It may rather consist of a (nonreflective) continuation and practical endorsement of social group norms, some of them moral norms. Thus, the individualistic parenting that *maybe* could distinguish human parenting from nonhuman animal parenting is a relatively young cultural aberration. Since communitarian parenting is not uniquely human (Hrdy 2009, ch. 6), excluding nonhuman animals from morality would mean biting the bullet of excluding humans to a highly implausible extent.

Moreover, there are examples of nonhuman animals educating their young morally, albeit nonreflectively. Nonhuman animal children need to learn group norms, how to behave properly towards others depending on their status, how to play fairly and so on (Andrews 2020a). Care behavior itself is partially learned, including parental skills (e.g. Champagne & Meaney 2001) and consolation behavior (Clay & de Waal 2013). Thus, the difference between human and nonhuman animal capacity for moral education of their young is gradual. Importantly, while the capacity for reflective commitment to morality may, indeed, set some practices of human caring apart from care in other animals, this does not prove that this second-order reflection is what distinguishes *moral* from *nonmoral* care. I suspect that this assumption rests on a conflation of the capacity for moral behavior with the capacity to consciously adhere to an ethical framework. However, we do not have to be care ethicists to care morally. To borrow an analogy from Andrews et al. (2018, 90), this would be akin to setting poetry as the benchmark for language capacities, and it would backfire in the human case as well. Ultimately, both Held and Noddings’ arguments point to features that may distinguish some forms of human caring from care in other animals, but they fail to prove that this distinction implies that only the former is moral.

## Caring animals

I have made the case that nonreflective care can be acknowledged as moral in a care ethics framework when it is motivated by a minimal form of empathy. This kind of care entails responsibility understood as prospective responsiveness. In this section I will take a look at the potential for and prevalence of moral care thus understood in nonhuman animals. Because care ethicists explicitly accept that moral concern emerges in the context of kin relationships, I will turn to our current scientific knowledge about parent-offspring relationships in nonhuman animals, and show that at least some of these cases fit the criteria for moral care. I also briefly outline how parental care in nonhuman animals shapes the capacity for forms of moral care outside this primary relationship, which likewise coincides with what care ethicists value about human parental care, i.e. that it produces moral beings. Lastly, I illustrate the notion of responsibility in nonhuman animals with some examples.

### Parental care as moral care in nonhuman animals

Parental care^7^ for offspring is widespread in nature. Across many species, infants need some level of care to survive their first days, months, or even years (Pianka 1970; Decety et al. 2016) argue that extensive parent-offspring relationships are one of the primary contexts for empathy to have evolved, because increased sensitivity to offspring needs boosts the quality and effectiveness of parental care and thus leads to increased fitness. This is supported by increasing evidence that empathy is “phylogenetically ancient, probably as old as mammals and birds” (de Waal 2008, 279). Likewise, Decety et al. (2016, 1) conclude that empathy is “common to humans and many animals,” with empathy understood as “the natural ability to perceive and be sensitive to the emotional states of others, coupled with a motivation to care for their well-being.” This is compatible with our concept of empathic care motivated by MME. Indeed, emotional contagion as a basic form of or precursor to more complex forms of empathy has been found in many nonhuman animals, for example in pigs (Reimert et al. 2015; Goumon & Špinka 2016), chimpanzees (Parr 2001), geese (Wascher et al. 2008), dogs (Huber et al. 2017; Quervel-Chaumette et al. 2016; van Bourg et al. 2020), mice (Langford et al. 2006; Jeon et al. 2010), rats (Knapska et al. 2006; Atsak et al. 2011), prairie voles (Burkett et al. 2016), and chickens (Edgar et al. 2011). Moreover, some nonhuman animals have been speculated to possess more complex forms of empathy that involve a degree of perspective-taking, e.g. cows (Ede et al. 2020, 7), cetaceans, some primates (see Pérez-Manrique & Gomila [2018] for a review), and elephants (Bates et al. 2008). Thus, many nonhuman animals possess the empathic capacities necessary for moral care in the minimal sense that I have defended.

For the case of social mammals, we know that parental care also has a strong effect on social development, and the embodied nature of parental care is key for this (Harlow 1958; Harlow et al. 1965). In fact, the effects of parental touch on development seem to generalize across mammal species in two major regards, as Monsó and Wrage (2021) point out: Firstly, parental touch has an *influence on emotional self-regulation*. It has an immediate soothing effect as well as a long-term effect on stress response, i.e. parental touch helps the infant regulate their arousal and predicts their capacity to self-regulate their stress response as an adult (Hertenstein et al. 2006). Secondly, parental touch has an *influence on the capacity to form attachments*. It is the crucial element that creates and maintains the bond between infant and primary caregiver, which, in turn, sets the stage for the capacity to form attachments more generally, and thus informs the occurrence and quality of future relationships (Feldman 2011; Hertenstein et al. 2006). Parental care thus shapes two capacities that are highly relevant for an individual’s social life (see Monsó & Wrage 2021).

This influence of parental care on emotional self-regulation and on the capacity to form attachments causally connects parental care to capacities of empathic care in social animals. To be able to care for another in these terms, one needs to be able to overcome a purely egoistical perspective, which requires the capacity to *self-regulate* (Monsó & Wrage 2021). Being overwhelmed by distress is counterproductive to empathic caring, because it prevents the individual from being able to pay attention to others and *empathize* with them, as demonstrated in rats by Ben-Ami Bartal et al. (2016), and in dogs by Sanford et al. (2018). Attention, in turn, can be further motivated by *attachment*. For example, paying attention to one’s child or partner and the readiness to care for them comes more naturally than paying attention to and caring for a neutral stranger (van Berlo et al. 2020). Indeed, prairie voles, for example, will console their partners but not strangers (Burkett et al. 2016), rats are more likely to help familiar conspecifics than unfamiliar ones (Ben-Ami Bartal et al. 2014), and familiarity modulates emotional responses to distress calls in cockatiels (Liévin-Bazin et al. 2018). Taken together, a being with low emotional self-regulation and no or unstable attachments will be less likely to be able and motivated to care. This is also evident in the care behavior of parentally deprived nonhuman animals: Orphaned bonobos, for instance, are less likely than mother-reared bonobos to console others in distress (Clay & de Waal 2013), and parentally deprived nonhuman animals show inhibited parental care towards their own offspring (e.g. in rhesus monkeys: Arling & Harlow 1967; e.g. in rodents: Champagne & Meaney 2001; Gonzalez et al. 2001; Kikusui et al. 2005). Taken together, empathic parental care in nonhuman animals is thus itself a moral behavior, but it is also a precondition for caring animals to develop as such. This corresponds to the care ethical idea that parental care produces moral beings who draw from their own experienced care to care for others.

### Empathic care beyond the parent-infant relationship: helping and consolation

To put the effect of parental care on nonhuman animals’ caring capacities into context, I want to briefly mention two forms of emotional care beyond the parent-infant relationship: helping and consolation. Both these behaviors may plausibly be shaped by experienced parental care (see above). First, helping is defined as intentional behavior to benefit another regardless of personal gain (Cronin 2012). While cognitively demanding considerations of fairness may sometimes play a motivating role in human helping, the literature on helping across species suggests that it has its origin in more basic emotional processes, namely empathy (Marsh et al. 2014). Empathic helping has been observed in bonobos (Melis 2018), chimpanzees (Yamamoto et al. 2012), dogs (towards humans: Sanford et al. 2018; van Bourg et al. 2020), dolphins (Park et al. 2013), elephants (Bates et al. 2008), humpback whales (Pitman et al. 2017), mice (Ueno et al. 2019), and rats (Ben-Ami Bartal et al. 2011; Carvalheiro et al. 2019).

Second, besides helping, another relatively well studied form of emotional care in nonhuman animals is consolation behavior, which is defined as an increase in affiliative contact towards a conspecific in distress (Burkett et al. 2016). It is usually studied as a post-conflict behavior, where a bystander approaches the loser of a fight and affiliates with them (e.g. de Waal & van Roosmalen 1979), or, in the lab, as a behavior in response to a conspecific who experienced an aversive stimulus (e.g. Burkett et al. 2016). Consolation is likewise a form of emotional care, as it is thought to be motivated by empathic processes and seems to have a calming effect on the recipient, thus meeting their needs by helping them cope with a stressful situation. Consolation is found in a range of nonhuman animals, including dogs (Quervel-Chaumette et al. 2016), dolphins (Yamamoto et al. 2015), corvids (Seed et al. 2007; Fraser & Bugnyar 2010), elephants (Plotnik & de Waal 2014), primates (de Waal & van Roosmalen 1979; Palagi et al. 2004; Cordoni et al. 2006; McFarland & Majolo 2012), and voles (Burkett et al. 2016).

The available data on helping and consolation as well as underlying empathic capacities in a range of nonhuman animals from rodents to great apes to some birds is substantial. This should make clear that I am not basing my case for the acknowledgment of caring animals on a rare occurrence in a few select, maybe especially human-like species.^8^ Indeed, it is not my goal to argue that some nonhuman animals are exceptionally similar to us and thus worthy of recognition by care ethics. Instead, I propose that the most compelling claims of care ethics regarding the phylogenetic and ontogenetic origins of morality naturally include many nonhuman animals. It is empirically untenable to anthropo-monopolize care, a capacity so fundamental to human and nonhuman animal social life.

### Responsible animals

I have argued that care ethics emphasizes a notion of responsibility that is an inherent feature of empathic care. Thus, all examples of empathic care in nonhuman animals are also examples of responsibility in nonhuman animals. I still want to highlight some that I find especially compelling. Foremost, responsibility is evident in the ways in which parents adapt their behavior to the abilities of their young, or generally the special consideration of adult group members towards infants, from appropriate play behavior to adjusting one’s traveling speed to assuming responsibility for orphans. Furthermore, the policing of group conflicts, holding watch over others when they rest, alerting others of or teaching them about dangers, sharing food, supporting ill, injured, or disabled group members are instances of nonhuman animals taking responsibility.

One striking example of responsibility in the context of allo-parenting can be found in cooperatively breeding marmoset monkeys. These marmosets form groups in which related or non-related individuals support a breeding pair in raising their offspring. Brügger et al. (2018) investigated the hypothesis that helpers “help more if group members can witness their interactions with the immatures,” which would imply a motivation that has to do with social prestige or the “pay-to-stay” model, i.e. making visible contributions to the group to be granted continued membership (ibid., 1). They found that helper marmosets in fact *increase* their care behavior towards immatures when there are no other group members present. Brügger et al. interpret this behavior of the helpers “to reflect a genuine concern for the immatures’ well-being, which seems particularly strong when *solely responsible* for the immatures” (ibid., my emphasis).

The affidavits that were submitted by leading primatologists in support of the Nonhuman Rights Project around Steven Wise contending for the personhood of chimpanzees Tommy, Kiko, Hercules, and Leo, explicitly support this framing of care behavior as responsible behavior: Goodall (2015) describes the long-term responsibility of chimp mothers for their young, or the responsibility that is often assumed by adult male chimps for orphans (ibid., 6ff); Savage-Rumbaugh (2015) describes chimpanzees’ duties towards the group in the context of food sharing; she further writes: “In the case of chimpanzees and bonobos […], duties and responsibilities (and the moral imperatives they entail) are simply a part of everyday life.” (ibid., 6). Christophe Boesch speaks of chimpanzees’ “social obligations” in the context of defending territory, rescuing conspecifics, hunting, helping behavior, and alerting others of danger (Boesch 2015, 6ff). – The care behavior and thus responsibility of other hypersocial animals like cetaceans, elephants, and corvids has a similar richness. To name just a few examples, dolphins have been observed to adopt infants of different species (Carzon et al. 2019), and to help a dying conspecific stay afloat by propping them up (Park et al. 2013). Elephants have been observed to make concerted efforts to help infants across difficult terrain or out of a ditch (Bates et al. 2008, 216f), and they form alliances to rescue infants who have been kidnapped (ibid., 215f). All these behaviors consist of nonhuman animals actively taking on the task of answering to others’ dependence, and are thus instances of responsibility.

## Conclusion and outlook: what care do we owe to caring animals?

I have argued that we can acknowledge nonhuman caring animals and their intraspecific relationships of care as moral in a care ethics framework, and that this already includes the parental relationships of many species. My argument is based on empirical data on empathic care in nonhuman animals, and on a de-intellectualized notion of morality, centered on emotional motivation, that has been put forth in the animal morality debate. This non-traditional understanding of morality is not only highly compatible with a sentimentalist theory such as care ethics, but care ethics becomes more plausible if it renounces its intellectualization of care. By adopting a de-intellectualized notion of morality inclusive of caring animals, care ethics avoids intellectualistic and anthropocentric bias, accounts for paradigmatic forms of moral care, such as spontaneous acts of care, more adequately, and gains a more robust standing in relation to traditional moral theory.

The animal morality debate, too, stands to gain further from this connection to care ethics that I make. An account of morality that situates it in particular relationships instead of in the realm of abstract moral deliberation is more amenable to the idea of nonhuman animal morality from the start. Moreover, this opens the door for ethical reflection: Finding or assuming moral capacities in nonhuman animals should mean something for our treatment of these moral animals, which can be argued with care ethics. Interferences with nonhuman animals’ relationships and individual caring capacities are ubiquitous across all contexts of human-nonhuman animal interaction, especially in systems of use (see also Monsó et al. 2018; Cooke 2021). Nonhuman animals in labs, zoos, on farms, and in our homes are routinely deprived of stable social bonds and/or autonomy in navigating their social lives, two basic conditions for care. Moreover, human activity routinely disrupts wild nonhuman animal communities. If care is a value, the relationships of caring animals as I have described them possess this value. Thus, the prevention, disruption, manipulation, or instrumentalization of caring relationships likely constitute more than subjective welfare harms, and may need to be addressed as objective wrongs. In turn, meddling with individual nonhuman animals’ caring capacities may not even involve experiential harm, but it may still deprive them of leading a full moral life, from accessing other values like trust and friendship that are (only) accessible through care, or from the meaning that self-determined caring creates. This concerns nonhuman animals whose capacity to care is diminished, e.g. on purpose in animal experimentation or as a by-product of parental deprivation on farms, in zoos, the pet industry, as routine procedure in labs, and so on; but this also concerns nonhuman animals who are instrumentalized as caregivers and reduced to this capacity, for instance in dairy farming.

Care ethics compellingly shows that morality is neither rare nor exceptional, but that it is, in the form of care, a basic thread that runs through our lives. We should embrace the idea that this is true for many other animals as well, and that, hence, the world is more caring than we currently recognize, but also more vulnerable when we fail to recognize this.

## Footnotes

1 Care ethicists tend to avoid giving such a boiled down definition of care, so this is a first rough summary of the debate. I use the term “intentional” here in a very low-level sense, i.e. not involving metarepresentational capacities. Examples of intentional behaviors thus understood would be riding a bike on a familiar route, or playing a well-practiced sport, i.e. behaviors done with purpose but not necessarily reflectively. Many care ethicists understand intentional care to require a more cognitively complex version of intentionality, which is precisely the challenge for my argument. I return to the question how much (meta-)cognition care requires in Sects. 2 and 3.

2 These two volumes contain the earliest texts on the care ethical tradition in animal ethics, edited by two of its founders. Contributions range from criticism of animal rights theory to a call for sympathy and care for nonhuman animals, to the application of care ethics to the problem of wild animal suffering or the meat industry. Thus, they develop the idea that humans owe care to nonhuman animals, and that this can be the basis for animal ethics beyond individual rights.

3 I share Gruen’s (2015, 43f) reservations regarding the focus of sympathy (that is on negative circumstances, like the distress of the other) and her turn to empathy understood as “Einfühlung” in the sense of Theodor Lipps, the immediate sensitive perception of another’s emotional expression.

4 As stated before, I assume that intentional behavior can be nonreflective, e.g. like riding a bike on a familiar route or playing a well-practiced sport. Accounts of intentionality thus understood and attributed to animals have, for example, been put forth by Sebo (2017) and Wilcox (2019).

5 The idea that reasons can be nonreflective is also defended by Waller (1997) and Rowlands (2012, 90ff).

6 Thank you to Kristin Andrews for pointing this out to me.

7 I use the gender-neutral terms ‘parental care’ and ‘primary caregiver’ to refer to any primary caregiver(s), i.e. to include species with bi-parental, allo-parental, maternal, or paternal primary care.

8 Such an argument from the perspective of exceptionalism has been made for the case of care by Jaworska (2007), who proposes that some great apes may have “full moral standing” on account of their exceptional capacities for inter-specific care. However, even such an argument may need to acknowledge many more species than just great apes, since there is anecdotal evidence of inter-specific care in a range of species, e.g. of cross-species adoption in dolphins (Carzon et al. 2019) and capuchin monkeys (Izar et al. 2006), farmed and companion animals in sanctuaries routinely taking care of and forming friendships with heterospecifics, and the mutual caring relationships that nonhuman animals have with humans.
